# Association of transport time with adverse outcome in paediatric trauma

**DOI:** 10.1093/bjsopen/zrab036

**Published:** 2021-04-08

**Authors:** Helen Träff, Lars Hagander, Martin Salö

**Affiliations:** 1 Department of Clinical Sciences, Paediatrics, Lund University, Lund, Sweden; 2 Department of Paediatric Surgery, Skåne University Hospital, Lund, Sweden

## Abstract

**Background:**

It is unclear how the length of prehospital transport time affects outcome in paediatric trauma. This study evaluated the association of transport time from alarm to arrival at hospital with adverse outcome in paediatric trauma patients in Sweden.

**Methods:**

This was a retrospective study based on prospectively collected data from the Swedish trauma registry between 2012 and 2019 of children less than 18 years with major trauma (New Injury Severity Score (NISS) greater than 15). The primary outcome was 30-day mortality, and secondary outcomes were emergency interventions (e.g., chest tube or laparotomy) and low functional outcome (Glasgow Outcome Scale 2–3). Primary exposure was transport time from alarm to arrival at hospital. Co-variables in multivariable regressions were gender, age, ASA score before injury, injury intention, dominant injury type, NISS, Glasgow Coma Scale score, prehospital competence and hospital level.

**Results:**

Among 597 patients, 30-day mortality was 9.8 per cent, emergency interventions were performed in 34.7 per cent and low functional outcome was registered in 15.9 per cent. Median transport time was 51 (i.q.r. 37–68) minutes. After adjustment for patient, injury and hospital characteristics, no association between longer transport time and 30-day mortality, frequency of emergency interventions or lower functional outcome could be found. Treatment at a university hospital was associated with a lower risk for 30-day mortality (odds ratio 0.23 (95 per cent c.i. 0.08 to 0.68), *P* = 0.008).

**Conclusion:**

Longer transport time after major paediatric trauma was not associated with adverse outcome. Hence, it seems that longer transport distances should not be an obstacle against centralization of paediatric trauma care. Further studies should focus on the role of prehospital competence and other transport-associated parameters and their association with adverse outcome.

## Teaser

The aim of the study was to evaluate the association of transport time with adverse outcome in pediatric trauma patients in Sweden, using a prospective trauma registry including children with major trauma. The median transport time from alarm to hospital was 51 minutes, and among 597 patients 30-day mortality was 10%, emergency interventions were performed in 35% and low functional outcome was registered in 16%. After adjustment for possible covariates, the results showed that longer transport time after major pediatric trauma was not associated with a higher 30-day mortality, a higher rate of emergency interventions or lower functional outcome.

## Introduction

Traumatic injuries affect children globally in their everyday life. Minor injuries often result in full recovery, while major trauma can lead to numerous emergency interventions and various degrees of long-term disability, pain, and impact on quality of life, career and income for the children and their families. Mortality after traumatic injuries is substantial, with injuries and violence accounting for almost one million global deaths in children under the age of 18 annually^1^^,^^2^. Through extensive preventive measures, Sweden has, in the past decades, efficiently reduced injuries in the child population by 75 per cent^3^, yet injuries still account for one in five deaths and one in six cases of hospitalizations^4^. Despite total injury prevention being the ultimate goal, studies suggest that well structured trauma care can decrease mortality risk after trauma^5^^,^^6^.

A renowned concept in trauma care, attributed to R. Adams Cowley, is ‘the golden hour’. The term refers to the principle that the risk of adverse outcome – a higher morbidity and mortality – would increase if the patient were not given correct emergency medical care within 60 minutes from encountering trauma^7^. A number of studies have thereafter presented results supporting the golden hour^8–12^, while others report inconclusive or contradictory results in both paediatric and adult trauma patients^13–16^. A recent Swedish trauma study including patients of all ages found no association between longer transport time and adverse outcome, although it did not specifically investigate the paediatric population^17^. Despite extensive previous research investigating a suggested correlation between prehospital time and adverse outcome after trauma, the results remain inconclusive and the definite impact of transport time on paediatric trauma patients is still unclear^18^. Another vital question when discussing trauma care is the ongoing centralization of trauma centres and surgical subspecialization, and the resulting differences in paediatric trauma capacity between hospitals. A higher level of trauma care is shown to result in improved outcomes, making it important to, on one hand, consider the centralization of trauma care, against, on the other, the aspect of longer transport time from trauma to arrival at the treating hospital^19^.

The aim of this study was to investigate how prehospital transport time was associated with adverse outcome after paediatric trauma in Sweden. The prospective Swedish national trauma registry offers an opportunity to perform such time analyses in the paediatric population, in regard to mortality, emergency interventions and functional outcome. The purpose of this study was to contribute with a better understanding of the advantages and disadvantages of trauma care centralization at a national level.

## Method

### Study design and setting

This was a retrospective study on prospectively collected register-based data of all cases of paediatric trauma between January 2012 to June 2019 registered in the Swedish National Quality Registry for Trauma (SweTrau).

### Inclusion and exclusion criteria

Any child less than 18 years of age with major trauma (New Injury Severity Score (NISS) greater than 15) was eligible for inclusion. Patients were excluded where date of birth was uncertain or falsely registered, as were those with missing data on transport time from alarm until arrival at hospital. Patients with secondary transport to another hospital or with missing data on transport modality were also excluded.

### Data sources

SweTrau is a prospective national register of trauma in Sweden. From the 52 hospitals in Sweden with a round-the-clock emergency clinic, 48 hospitals (92 per cent) are connected to SweTrau. The register has an opt-out approach, meaning that the participants are included unless they ask to be excluded. SweTrau captures major trauma caused by road traffic incidents, falls and other external trauma for both adults and children. Variables are recorded according to the Utstein trauma template for uniform reporting of data following major trauma^20^. Procedures are classified according to lists from the Swedish National Board of Health and Welfare^21^. The SweTrau register had a national coverage of around 65 per cent in 2018, with generally higher coverage at hospitals treating children^22^.

### Outcome variables

The primary outcome was 30-day mortality rate. Secondary outcomes were emergency interventions and functional outcome. Mortality at 30 days included deaths in hospital and after discharge. Emergency interventions were defined as thoracotomy, laparotomy, pelvic packing, limb revascularization, interventional radiology, craniotomy, intracranial pressure device insertion, chest tube, external fracture fixation, major fracture surgery and wound revision. Functional outcome was measured with the Glasgow Outcome Scale (GOS)^23^ and categorized into two groups: persistent vegetative state/severe disability (GOS 2–3) and moderate disability/good recovery (GOS 4–5). Deceased patients (GOS 1) were not included in the analyses on functional outcome.

### Primary exposure and potential confounders

Primary exposure was transport time from alarm until arrival at the hospital. Transport time was used as a continuous variable for the main analyses. In *Table 1*, median transport time was used as a cut-off to define short *versus* long transport time. Independent variables were gender, age, ASA physical status classification prior to the trauma incident, injury intention, mechanism of injury, dominant injury type (blunt or penetrating), NISS (16–75), Glasgow Coma Scale (GCS) score upon arrival at trauma scene (3–15), prehospital competence (a physician included in the prehospital resuscitation team or not) and receiving hospital (university or non-university hospital). Injury intention was defined as self-inflicted (suspected suicide, incomplete suicide attempt or injury attempt) or non-self-inflicted (unintentional accident or suspected assault). Mechanism of injury was defined as traffic-related injuries (including motor vehicles, motorcycles, bicycles and pedestrians), gunshot/stab injuries, injuries from blunt objects, high- and low-energy falls and explosion injuries. Mechanism of injury was divided into traffic- or non-traffic-related injuries in the univariable regression analyses, and was excluded in the multivariable regression analyses since the variable was considered too closely related to dominant injury type. Prehospital systolic blood pressure and respiratory rate at arrival at trauma scene were recorded in SweTrau as either a numeric value, or categories according to the Revised Trauma Score (RTS)^24^. All registered numeric values were analysed and age-standardized^25^. Age-standardized numeric values and RTS categories were categorized as either normal or non-normal values. Low systolic blood pressure and low/high respiratory rate were defined as non-normal.

**Table 1:** Variable distribution and comparison in paediatric trauma patients in Sweden, 2012–2019, divided into two groups: short transport time and long transport time

	**Short transport time** (<51 min)	**Long transport time** (>51 min)	*P*
	(*n* = 304)	(*n* = 293)	
**Gender**			0.554^§^
Female	105 (34.5)	108 (36.9)	
Male	199 (65.5)	185 (63.1)	
**Age (years)**			
Median (i.q.r.)	14 (9–16)	14 (9–16)	0.649
Mean(s.d.)	12.0 (5.2)	12.3 (4.8)	0.339
**ASA before injury**			0.767^§^
1	276 (92.6)	267 (92.1)	
2	19 (6.4)	21 (7.2)	
3	2 (0.7)	2 (0.7)	
4	1 (0.3)	0 (0)	
5	0 (0)	0 (0)	
**Injury intention**			0.045^§^
Non self-harm	280 (93.3)	281 (96.9)	
Self-harm	20 (6.7)	9 (3.1)	
**Mechanism of injury**			0.001^§^
Traffic-related^*^	139 (45.7)	161 (54.9)	
Penetrating objects^†^	29 (9.5)	11 (3.8)	
Blunt objects	18 (5.9)	17 (5.8)	
Falls^‡^	96 (31.6)	65 (22.2)	
Explosions	1 (0.3)	1 (0.3)	
Others	21 (6.9)	38 (13.0)	
**Dominant injury type**			0.026^§^
Blunt	272 (90.1)	278 (94.9)	
Penetrating	30 (9.9)	15 (5.1)	
**NISS**			
Median (i.q.r.)	22 (17–34)	22 (17–29)	0.026
Mean(s.d.)	28.4(14.6)	25.6(11.8)	0.011
**GCS sum at trauma scene**			
Median (i.q.r.)	15 (14–15)	13 (4–15)	<0.001
Mean(s.d.)	14.0(2.3)	10.2(5.1)	<0.001^#^
**Prehospital competence**			0.001
No physician present	275 (91.1)	240 (81.9)	
Physician field care	27 (8.9)	53 (18.1)	
**Hospital level**			<0.001^§^
University hospital	193 (63.5)	129 (44.2)	
Non-university hospital	111 (36.5)	163 (55.8)	

Values presented as the absolute number and percentage of patients unless indicated otherwise. The respective percentages are calculated from the total number of patients with registered values on the specific variable, missing values are not reported.

* Accidents involving motor vehicles, motorcycles, bicycles, other vehicles and pedestrians;

† gunshot or stab injuries;

‡ low- and high-energy falls.

§ NISS: New Injury Severity Score; GCS: Glasgow Coma Scale. χ^2^ test;

¶ Mann–Whitney *U* test; #independent samples *t*-test

### Statistical analysis

Categorical values were reported as the absolute numbers and percentages. Continuous data were reported as median with range and interquartile range (i.q.r.). When comparing two groups, χ^2^ test was used for categorical variables, and Mann–Whitney *U* test for continuous variables. Univariable and multivariable logistic regression models were presented as odds ratios (OR) with 95 per cent confidence intervals. Statistical power did not allow for the inclusion of all relevant potential confounders at the same time, and the multivariable regression was therefore performed in three different models with co-variables for patient characteristics, trauma characteristics and clinical presentation, respectively. The ‘Patient characteristics’ model included the following independent variables: gender, age and ASA score. The ‘Trauma characteristics’ model included injury intention and dominant injury type. The ‘Clinical presentation’ model included NISS, GCS score, prehospital competence and hospital level. Sensitivity analyses were performed with assessment for confounding by prehospital systolic blood pressure and respiratory rate against the full multivariable model. IBM SPSS Statistics for Macintosh, version 26.0 (IBM Corp., Armonk, N.Y., USA), was used for the calculations. Statistical significance was set to *P* < 0.050. This study had an ethical permit from the regional Ethical committee (reg. no 2018/772).

## Results

From the original group of 876 patients, exclusion was made due to missing data on transport time (246 patients, 28.1 per cent), missing value of age (25 patients, 2.9 per cent), duplicates (5 patients, 0.6 per cent) and missing data on transport type/secondary transport (3 patients, 0.3 per cent), leaving 597 patients (68.2 per cent) included in the study (*Table S1*). Of these, 64.3 per cent were boys with a median age of 14 (9–16) years. The dominant injury type was blunt trauma (92.4 per cent). Penetrating injuries were more common among the older children (80.0 per cent at least 14 years old). The most common trauma mechanism was high-energy falls (25.0 per cent) followed by motor vehicle accidents (19.8 per cent). Patients were transported by ground (86.6 per cent) or helicopter (13.4 per cent) ambulance. A minority had a physician included in the prehospital resuscitation team (13.4 per cent). The patients were treated at 35 hospitals, and 54.0 per cent of the children were treated at any of the seven university hospitals.

The mortality rate at 30 days was 9.8 per cent (56 patients), with equal gender distribution (53.6 per cent boys, 46.4 per cent girls). The highest mortality rates were found among the older children (58.9 per cent at least 14 years old). Day of death following trauma was registered in 52 of the non-survivors (92.9 per cent), with a median of 0 (range 0–6, i.q.r. 0–1) days. Among 33 patients where day of death was the same as the day of trauma, 24 (72.7 per cent) patients suffered from an injury-related prehospital cardiac arrest.

Emergency interventions were performed in 34.7 per cent of the included patients. The leading type of interventions performed were chest tubes (6.4 per cent) and craniotomies (4.9 per cent), followed by intracranial pressure device insertions (4.6 per cent), external fracture fixation (3.7 per cent) and major fracture surgery (3.7 per cent). Among survivors with a registered functional outcome after the trauma, 15.9 per cent had persistent vegetative state/severe disability (GOS 2–3) and 84.1 per cent had moderate disability/good recovery (GOS 4–5).

Transport time from alarm to arrival at hospital showed a median time of 51 (range 9–512, i.q.r. 37–68) minutes. Approximately one third (207) of the patients had a reported transport time exceeding 1 hour. Children with shorter transport time (51 minutes or less, 304 patients) more often had injuries resulting from self-harm, penetrating injuries, were more likely to be treated at a university hospital and reported a different distribution of NISS than children with longer transport time (greater than 51 minutes, 293 patients) (*Table 1*). Within the group with shorter transport time, the outcome was more often fatal compared with the group with longer transport time (*Table 2*). Time from trauma event to alarm was registered in 515 (86.3 per cent) of the patients, with a median time of 3 (i.q.r. 1–5) minutes.

**Table 2:** Outcome variables distribution and comparison in paediatric trauma patients in Sweden, 2012–2019, divided into two groups: short transport time and long transport time

	**Short transport time** (<51 min)	**Long transport time** (>51 min)	P*
	(*n* = 304)	(*n* = 293)	
**30-day mortality**			<0.001
Non-survivors	42 (14.1)	14 (5.1)	
Survivors	256 (85.9)	262 (94.9)	
**Emergency interventions**			0.654
Yes	107 (35.5)	98 (33.8)	
No	194 (64.5)	192 (66.2)	
**Functional outcome**			0.272
GOS 2–3	45 (17.7)	37 (14.2)	
GOS 4–5	209 (82.3)	224 (85.8)	

Values presented as the absolute number and percentage of patients. The respective percentages are calculated from the total number of patients with registered values on the specific variable, missing values are not reported. GOS: Glasgow Outcome Scale. *χ^2^ test.

**Fig. 1:**
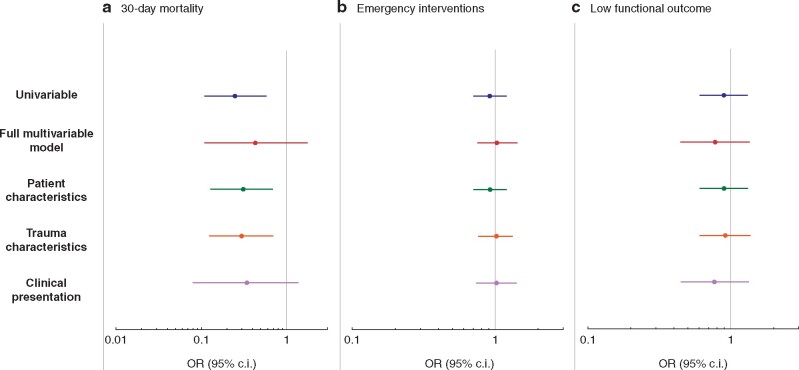
Univariable and multivariable regression analysis of transport time from alarm to arrival at hospital in paediatric trauma patients in Sweden, 2012–2019 **a** 30-day mortality. **b** Emergency interventions. **c** Low functional outcome. Full multivariable model: adjusted for gender, age, ASA classification before the trauma, injury intention, dominant injury type, New Injury Severity Score (NISS), Glasgow Coma Scale (GCS) score, prehospital competence and hospital level. Patient characteristics: adjusted for gender, age and ASA classification before the trauma. Trauma characteristics: adjusted for injury intention and dominant injury type. Clinical presentation: adjusted for NISS, GCS score, prehospital competence and hospital level. OR, odds ratio; c.i., confidence interval.

In the univariable regression analysis, a longer transport time was inversely associated with 30-day mortality rate (OR for death 0.25 per hour (95 per cent c.i. 0.11 to 0.58), *P* = 0.001). No association was found between longer transport time and emergency interventions or lower functional outcome (*Table 3*). After adjustment in multivariable analysis, the inverse association between longer transport time and 30-day mortality rate was no longer found. The results on frequency of emergency interventions and functional outcome remained largely unaltered after adjustment in multivariable analysis (*Fig. 1a–c*). Defining transport time in categories of 15 or 30 minute instead of hours did not affect the results. Further, the lack of association between longer transport time and adverse outcome remained in sensitivity analyses with prehospital systolic blood pressure and respiratory rate included in the full multivariable model (*Table S2*). Sensitivity analyses on the full multivariable model, excluding patients with prehospital cardiac arrest where day of death was the same as the day of trauma, were also performed with no association between longer transport time and 30-day mortality rate (OR 0.33 (95 per cent c.i. 0.05 to 2.18), *P* = 0.248) or frequency of emergency interventions (OR 1.05 (95 per cent c.i. 0.76 to 1.45), *P* = 0.760) found.

**Table 3:** Univariable regression analyses for 30-day mortality, emergency interventions and low functional outcome (Glasgow Outcome Scale 2–3) among 597 paediatric trauma patients

	30-day mortality	Emergency interventions	Low functional outcome (GOS 2–3)
**Time alarm–hospital (hours)**	0.25 (0.11–0.58)	0.92 (0.71–1.20)	0.90 (0.61–1.32)
	***P* = 0.001**	*P* = 0.556	*P* = 0.575
**Gender (boys)**	0.61 (0.35–1.06)	1.07 (0.75–1.53)	1.69 (0.99–2.87)
	*P* = 0.080	*P* = 0.697	*P* = 0.055
**Age (years)**	0.98 (0.93–1.04)	1.07 (1.03–1.11)	1.04 (0.99–1.10)
	*P* = 0.517	***P* < 0.001**	*P* = 0.122
**ASA before injury**	0.46 (0.12–1.81)	0.97 (0.57–1.65)	0.84 (0.38–1.88)
	*P* = 0.269	*P* = 0.906	*P* = 0.676
**Injury intention (self-harm)**	1.78 (0.59–5.37)	2.45 (1.15–5.20)	3.52 (1.49–8.36)
	*P* = 0.304	***P* = 0.020**	***P* = 0.004**
**Mechanism of injury (traffic-related)**	0.83 (0.48–1.44)	0.95 (0.68–1.34)	0.76 (0.47–1.22)
	*P* = 0.502	*P* = 0.772	*P* = 0.257
**Dominant injury type (penetrating)**	3.55 (1.68–7.49)	4.53 (2.34–8.76)	0.51 (0.15–1.71)
	***P* = 0.001**	***P* < 0.001**	*P* = 0.274
**NISS**	1.14 (1.11–1.17)	1.05 (1.03–1.06)	1.10 (1.08–1.13)
	***P* < 0.001**	***P* < 0.001**	***P* < 0.001**
**GCS score at trauma scene**	0.99 (0.93–1.06)	0.98 (0.94–1.02)	0.96 (0.91–1.01)
	*P* = 0.865	*P* = 0.379	*P* = 0.104
**Prehospital competence (physician field care)**	2.49 (1.28–4.82)	2.49 (1.53–4.06)	3.81 (2.13–6.82)
	***P* = 0.007**	***P* < 0.001**	***P* < 0.001**
**Hospital level (university hospital)**	1.32 (0.75–2.34)	1.95 (1.38–2.77)	1.39 (0.85–2.25)
	*P* = 0.336	***P* < 0.001**	*P* = 0.187

Values are odds ratios with 95% confidence intervals. GOS, Glasgow Outcome Scale; NISS, New Injury Severity Score; GCS, Glasgow Coma Scale. Univariable logistic regression analyses presented as odds ratios with 95% confidence intervals.

The multivariable analysis showed that patients treated at a university hospital had a lower risk for 30-day mortality compared with the patients treated at a non-university hospital (OR 0.23 (95 per cent c.i. 0.08 to 0.68), *P* = 0.008). Further, the results revealed a greater need for emergency interventions among older patients (OR 1.07 (95 per cent c.i. 1.02 to 1.12), *P* = 0.003), patients presenting with predominantly penetrating trauma (OR 2.82 (95 per cent c.i. 1.31 to –6.07), *P* = 0.008) as well as patients treated at a university hospital (OR 1.74 (95 per cent c.i. 1.14 to 2.68), *P* = 0.011). A lower functional outcome was found among patients where the resuscitation team had a higher prehospital competence (OR 2.98 (95 per cent c.i. 1.38 to 6.48), *P* = 0.006). A higher NISS was associated with increased risk for all three outcomes (*P* < 0.001 for all) (*Table 4*).

**Table 4:** Multivariable regression analyses for 30-day mortality, emergency interventions and low functional outcome (Glasgow Outcome Scale 2–3) among 597 paediatric trauma patients

	30-day mortality	Emergency interventions	Low functional outcome (GOS 2–3)
**Time alarm–hospital (hours)**	0.43 (0.11–1.75)	1.03 (0.75–1.42)	0.78 (0.45–1.35)
	*P* = 0.241	*P* = 0.839	*P* = 0.372
**Gender (boys)**	0.65 (0.24–1.76)	1.10 (0.71–1.68)	1.62 (0.86–3.08)
	*P* = 0.400	*P* = 0.679	*P* = 0.139
**Age (years)**	0.99 (0.90–1.09)	1.07 (1.02–1.12)	1.03 (0.97–1.10)
	*P* = 0.791	***P* = 0.003**	*P* = 0.354
**ASA before injury**	0.13 (0.01–18.84)	0.76 (0.38–1.49)	0.90 (0.38–2.16)
	*P* = 0.424	*P* = 0.420	*P* = 0.817
**Injury intention (self-harm)**	1.07 (0.20–5.74)	2.34 (0.88–6.26)	2.74 (0.81–9.33)
	*P* = 0.935	*P* = 0.090	*P* = 0.107
**Dominant injury type (penetrating)**	3.48 (0.86–14.12) *P* = 0.081	2.82 (1.31–6.07) ***P* = 0.008**	0.25 (0.05–1.15) *P* = 0.074
**NISS**	1.17 (1.12–1.21)	1.05 (1.03–1.06)	1.09 (1.05–1.12)
	***P* < 0.001**	***P* < 0.001**	***P* < 0.001**
**GCS score at trauma scene**	1.11 (0.97–1.26)	0.99 (0.94–1.03)	0.98 (0.92–1.05)
	*P* = 0.127	*P* = 0.549	*P* = 0.621
**Prehospital competence (physician field care)**	1.35 (0.41–4.51)	1.76 (0.96–3.26)	2.98 (1.38–6.48)
	*P* = 0.625	*P* = 0.070	***P* = 0.006**
**Hospital level (university hospital)**	0.23 (0.08–0.68)	1.74 (1.14–2.68)	1.11 (0.60–2.05)
	***P* = 0.008**	***P* = 0.011**	*P* = 0.729

Values are odds ratios with 95% confidence intervals. GOS: Glasgow Outcome Scale; NISS: New Injury Severity Score; GCS: Glasgow Coma Scale. Multivariable logistic regression analyses presented as odds ratios with 95% confidence intervals.

## Discussion

This national register-based study of paediatric trauma investigated the impact of transport time on adverse outcome. The main finding in this study was that a longer transport time from alarm to arrival at hospital was not associated with a higher 30-day mortality rate, a higher amount of emergency interventions, or with lower functional outcome after major paediatric trauma.

The median transport time was 51 minutes, showing that the prehospital time takes up a great amount of the ‘golden hour’ of paediatric trauma care in Sweden. However, evaluation of the golden hour can be challenging as the acute situation when managing trauma patients may affect the precision of the time intervals reported, resulting in potentially inaccurate estimations^26^. Despite the rather long median transport time, no subsequent negative effect on the outcomes could be shown. This could be because, despite injuries leading to a high NISS, the patient was in a stable condition and could be transported in a safer and slower manner. Another explanation could be that adverse outcomes are affected by prehospital interventions and acute stabilization of a critically ill patient, rather than the transport time. Transport time from alarm to arrival at hospital showed a broad range, 9–512 minutes, and an interquartile range of only 37–68 minutes. The full range could be explained by a small number of outliers. In cases where a first ambulance arrives at the trauma scene and does not collect the patient, later requiring a second ambulance to come to the scene, a longer transport time from the first received alarm to arrival at hospital is reported.

Perhaps a higher mortality rate than 9.8 per cent would have been found with a longer follow-up time, since long-term survival among patients in a persistent vegetative state (GOS 2) remains unknown. Lower mortality rates were found in other studies^15^^,^^16^^,^^27^^,^^28^. Allen and colleagues reported a mortality rate of 3.6 per cent, however they had no lower inclusion limit of Injury Severity Score (ISS), consequently including paediatric patients with minor trauma. According to a report from the Swedish National Board of Health and Welfare, 49 children died following traumatic injuries in 2016^4^. In the present study, 56 children died between 2012 and 2019, resulting in a lower number of deaths per year than in the mentioned report. The difference could be ascribed to a lower national coverage, or the fact that patients clearly deceased at the trauma scene were never transported to a hospital and hence not included in SweTrau. Prior studies on paediatric trauma have, in line with this study, reported a predominance of boys^2^^,^^16^^,^^27^. However, the higher mortality rate among boys in other studies^2^^,^^4^^,^^28^ was not found in this study.

One third of the included patients underwent one or more emergency interventions after the trauma. Interventions were more frequently performed on patients treated at a university hospital compared with a non-university hospital, possibly pointing to effects of the centralization of trauma care. Non-university hospitals had a higher 30-day mortality rate than university hospitals, and this could point to undertreatment of patients at non-university hospitals, perhaps in relation to minor practical experience of emergency trauma interventions of the surgeon as another consequence of centralization.

A lower functional outcome was found among patients where the resuscitation team included a physician (hence accessed higher prehospital competence). Despite adjustment for trauma severity, this could possibly be explained by a triage process assigning a physician to the team when receiving an alarm of a severely injured child. Notably, low functional outcome (GOS 2–3) was found among 15.9 per cent of the survivors, with physical and/or cognitive complications requiring daily support^23^. The quality of life after the trauma was beyond the scope of this study, but one can assume that the incident has caused negative effects on everyday life for these children. While moderate disability (GOS 4) is not defined as the lower functional outcome in this study, these patients can also suffer from varying degrees of complications.

A foundation of properly trained personnel and adequate equipment should be found in both university and non-university hospitals, in order to provide acute care to stabilize severely injured trauma patients until, if needed, transport to a higher level of care can occur^29^. Previous studies have shown a lower mortality rate after trauma when treated at a level I trauma centre compared with non-trauma centres^5^. Despite the fact that none of the university hospitals in Sweden is considered to be a complete level I trauma centre, the results showed a lower mortality rate when the trauma care was provided in university hospitals. These findings are in line with a previous Swedish study including both paediatric and adult trauma patients, and argue for continued efforts at regionalization^17^.

There are some limitations to this study. The intense situation when managing a severely injured child could affect the precision of the reported time intervals. Incorporation of time from trauma to alarm in the multivariable regression model may have been of value when analysing the outcomes. Another limitation is that SweTrau lacked documentation regarding the conveyed transport distance. This would have enabled analysis of the transport time in relation to transport speed, since potential biases are the possibility that less sick patients are being transported less urgently, or that the prolonged transport time in fact is a consequence of more extensive prehospital interventions (‘stay and play’). Further, cause of death is not documented in the registry, but could have given valuable information as to the influence of transport time on mortality. Another possible limitation is the use of the Glasgow Outcome Scale (GOS) as a measure of disability. Despite it being time-effective and considered a standard outcome measure after trauma, its sensitivity in a paediatric trauma population has been debated^30^^,^^31^.

Paediatric trauma includes a wide panorama of injuries with different needs and possibilities. The acute care given includes an extensive amount of investigations and interventions in hope of survival and as few long-term complications as possible. To the knowledge of the authors, research is very limited on the time aspect of paediatric trauma care in Scandinavia in recent times. In an era when new trauma centres are developed, it is important to have a foundation of revised research results when establishing new guidelines and routines.

A correlation between longer transport times and adverse outcome after paediatric trauma could not be shown. Despite the interventions performed, a relatively large proportion of the children suffered from severe to persistent disability or did not survive after their trauma. Further studies on paediatric trauma care are needed to provide a greater knowledge regarding the potential impact of transport time after trauma, but also the effect of the interventions performed and the outcomes for this group of patients.

## Supplementary Material

zrab036_Supplementary_Data

## Notes

A previous version of the abstract was accepted for presentation at the European Pediatric Surgical Association Congress 2020. The congress did not take place due to the Covid-19 outbreak.
