# Supplementary material for: Association of transport time with adverse outcome in paediatric trauma
Source: BJS Open. 2021 Apr 8;5(3):zrab036. doi: 10.1093/bjsopen/zrab036 (PMC8105622; doi:10.1093/bjsopen/zrab036)
Supplement: zrab036_Supplementary_Data [file zrab036_supplementary_data.docx]

**SUPPLEMENTARY TABLES**

**Table 1.** Variable distribution and comparison in pediatric trauma patients in Sweden, 2012–2019, divided into two groups; included patients and total cohort (including patients with missing data on transport time).

|  | **Included patients**  **(n=597)** | **Total cohort**  **(n=843)** | **p-value** |
| --- | --- | --- | --- |
| **Gender**  Female  Male | 213 (35.7%)  384 (64.3%) | 320 (38.0%)  523 (62.0%) | 0.377 |
| **Age** (years) | 14 (9–16) | 14 (8–16) | 0.206 |
| **ASA before injury**  1  2  3  4  5 | 543 (92.3%)  40 (6.8%)  4 (0.7%)  1 (0.2%)  0 (0%) | 758 (91.3%)  64 (7.7%)  7 (0.8%)  1 (0.1%)  0 (0%) | 0.895 |
| **Injury intention**  Non self-harm  Self-harm | 561 (95.1%)  29 (4.9%) | 794 (95.3%)  39 (4.7%) | 0.839 |
| **Mechanism of injury**  Traffic-related^a^  Penetrating objects^b^  Blunt objects  Falls^c^  Explosions  Others | 300 (50.3%)  40 (6.7%)  35 (5.9%)  161 (27.0%)  2 (0.3%)  59 (9.9%) | 395 (47.0%)  45 (5.3%)  72 (8.6%)  249 (29.6%)  2 (0.2%)  78 (9.3%) | 0.271 |
| **Dominant injury type**  Blunt  Penetrating | 550 (92.4%)  45 (7.6%) | 791 (94.1%)  50 (5.9%) | 0.224 |
| **NISS** | 22 (17–33) | 22 (17–33) | 0.889 |
| **GCS sum at trauma scene** | 15 (11–15) | 15 (11–15) | 0.999 |
| **Prehospital competence**  No physician present  Physician field care | 515 (86.6%)  80 (13.4%) | 591 (87.9%)  81 (12.1%) | 0.458 |
| **Hospital level**  University hospital  Non-university hospital | 322 (54.0%)  274 (46.0%) | 538 (63.9%)  304 (36.1%) | **<0.001** |
| **30-day mortality**  Non-survivors  Survivors | 56 (9.8%)  518 (90.2) | 61 (7.5%)  751 (92.5%) | 0.139 |
| **Emergency interventions**  Yes  No | 205 (34.7%)  386 (65.3%) | 301 (36.0%)  535 (64.0%) | 0.608 |
| **Functional outcome**  GOS 2–3  GOS 4–5 | 82 (15.9%)  433 (84.1%) | 158 (21.0%)  596 (79.0%) | **0.025** |

Values presented as the absolute number and percentage of patients, n (%); and as median (IQR). Percentages are calculated from the total number of patients with registered values on the specific variable, missing values are not reported. P-values presented per request from reviewers and editor. ASA: American Society of Anesthesiologists; IQR: Interquartile range; NISS: New Injury Severity Score; GCS: Glasgow Coma Scale. ^a^Accidents involving motor vehicles, motorcycles, bicycles, other vehicles and pedestrians; ^b^Gunshot or stab injuries; ^c^Low and high energy falls

**Table 2.** Assessment for confounding by prehospital systolic blood pressure and prehospital respiratory rate against the full multivariate regression analysis of transport time and its association with 30-day mortality, emergency interventions and low functional outcome

|  | **30-day mortality**  OR [95% CI] | **Emergency interventions**  OR [95% CI] | **Low functional outcome (GOS 2-3)**  OR [95% CI] |
| --- | --- | --- | --- |
| **Time alarm–hospital** (Hours) | 0.62 [0.15–2.49]  p =0.497 | 0.83 [0.56–1.23]  p =0.350 | 0.94 [0.56–1.58]  p =0.820 |
| **Gender**  (Boys) | 0.68 [0.24–1.87]  p =0.449 | 1.22 [0.77–1.95]  p =0.397 | 1.44 [0.74–2.78]  p =0.280 |
| **Age** (Years) | 1.00 [0.90-1.12]  p =0.994 | **1.06 [1.01–1.11]**  **p =0.028** | 1.05 [0.98–1.12]  p =0.200 |
| **ASA before injury** | 0.13 [0.00–29.11]  p =0.461 | 0.64 [0.31–1.34]  p =0.239 | 0.92 [0.38–2.23]  p =0.851 |
| **Injury intention**  (Self-harm) | 0.88 [0.16–4.91]  p =0.885 | 1.83 [0.65–5.15]  p =0.251 | 2.45 [0.65–9.18]  p =0.185 |
| **Dominant injury type**  (Penetrating) | 3.54 [0.90–13.91]  p =0.070 | **2.95 [1.29–6.76]**  **p =0.011** | 0.24 [0.05–1.21]  p =0.084 |
| **NISS** | **1.17 [1.12–1.22]**  **p <0.001** | **1.04 [1.02–1.06]**  **p <0.001** | **1.10 [1.06–1.14]**  **p <0.001** |
| **GCS score at trauma scene** | 1.04 [0.90–1.21]  p =0.575 | 1.01 [0.95–1.08]  p =0.791 | 0.96 [0.88–1.04]  p =0.316 |
| **Prehospital competence**  (Physician field care) | 1.38 [0.40–4.73]  p =0.606 | 1.57 [0.81–3.08]  p =0.185 | **2.91 [1.27–6.68]**  **p =0.012** |
| **Hospital level**  (University hospital) | **0.26 [0.08–0.81]**  **p =0.021** | **1.97 [1.24–3.15]**  **p =0.004** | 1.30 [0.68–2.50]  p =0.432 |
| **Prehospital systolic blood pressure**  (Low) | **0.10 [0.01–0.96]**  **p =0.046** | **2.27 [1.05–4.90]**  **p =0.037** | 0.51 [0.17–1.47]  p =0.210 |
| **Prehospital respiratory rate**  (Low/high) | 1.10 [0.35–3.40]  p =0.873 | 1.24 [0.75–2.05]  p =0.395 | 1.01 [0.50–2.05]  p =0.983 |

OR: Odds Ratio; CI: Confidence Interval; GOS: Glasgow Outcome Scale; ASA: American Society of Anesthesiologists; NISS: New Injury Severity Score; GCS: Glasgow Coma Scale.
